# Biomechanical and biological features of hyaluronic acid in combination with chondroitin and platelet rich plasma for regenerative medicine applications

**DOI:** 10.3389/fbioe.2025.1607469

**Published:** 2025-10-07

**Authors:** Valentina Vassallo, Celeste Di Meo, Antonella D’Agostino, Annalisa La Gatta, Donatella Cimini, Giuseppe Toro, Giovanni Iolascon, Maddalena Mastrogiacomo, Chiara Schiraldi

**Affiliations:** ^1^ Department of Experimental Medicine, University of Campania “Luigi Vanvitelli”, Naples, Italy; ^2^ Department of Life Sciences, Health and Health Professions, Link Campus University, Rome, Italy; ^3^ Department of Environmental, Biological and Pharmaceutical Sciences and Technologies, University of Campania “Luigi Vanvitelli”, Caserta, Italy; ^4^ Department of Medical and Surgical Specialties and Dentistry, University of Campania “Luigi Vanvitelli”, Naples, Italy; ^5^ Department of Internal Medicine and Medical Specialties (DIMI), University of Genoa, Genoa, Italy

**Keywords:** viscosupplementation, glycosaminoglycans, hyaluronic acid, biofermentative chondroitin, platelet-rich plasma, osteoarthritis, human primary pathological chondrocytes, time-lapse video-microscopy

## Abstract

Currently, one of the most common treatments for osteoarthritis (OA) is viscosupplementation using intra-articular injectable gels, often based on glycosaminoglycans (GAGs), specifically hyaluronic acid (HA) and, in some cases, chondroitin sulfate (CS). Recently, the potential benefits of pharma-grade biofermentative unsulfated chondroitin (BC) have been established, particularly when combined with high molecular weight hyaluronan (HHA). Beyond GAGs, platelet-rich plasma (PRP) has also been reported to have beneficial effects, although many clinical studies lack proper control groups. The aim of this study was to perform a comparative analysis of injectable formulations based on BC combined with HHA (HHA/BC), both alone and in combination with PRP, to evaluate their rheological and biological properties. Flow curves and mechanical spectra of HHA/BC and HHA/BC+PRP were obtained to assess their viscoelastic behavior in relation to synovial fluid characteristics. Then, these two formulations were tested on human chondrocytes isolated from OA joints to investigate their functional role *in vitro* on specific biochemical pathways. Additionally, a chondrocyte monolayer scratch assay was performed to evaluate their repair potential using time-lapse video-microscopy. Finally, chondrocytes were cultured in GAG-based gels on transwell inserts for 14 days to mimic a 3D-like *in vitro* environment. HHA/BC+PRP exhibited a consistent rheological profile, supporting its potential application in intra-articular injections. Furthermore, the maintenance of cell phenotype was confirmed through the analysis of collagen type 2A1 (COL2A1) and aggrecan (ACAN) expression. The addition of PRP further enhanced the ability of GAGs to reduce specific pro-inflammatory and degradative OA-related markers (e.g., interleukin IL-6, NF-κB, metalloprotease MMP-13, and cartilage oligomeric matrix protein COMP-2). Both HHA/BC and HHA/BC+PRP similarly prompted scratch repair. Overall, these outcomes provide deeper insights into the biochemical and biological properties of these innovative injectable formulations, highlighting their potential application in OA management.

## 1 Introduction

Nowadays, Osteoarthritis (OA) represents a serious global public health problem. It is a chronic and progressive joint disease; it has been estimated that the incidence of OA will increase over the human lifespan, especially due to the rising prevalence of obesity (Cross et al., 2014; Bennell et al., 2017). Moreover, no definitive cure for OA is available, so current treatments are focused on symptom remission, joint function recovery and above all pain relief (Fonsi et al., 2019). According to the 2013 guidelines of the American Academy of Orthopedic Surgeons (AAOS), patients are initially required to follow a healthy lifestyle, physical therapy, weight control (balanced diet), regular exercise and neuromuscular education (Ferreira et al., 2024; Raeissadat et al., 2021).

The second therapeutic approach includes the oral administration of analgesics and non-steroidal anti-inflammatory drugs (NSAIDs) (Kilincoglu et al., 2015; Smith et al., 2020). Unfortunately, constant and prolonged use of NSAIDs leads to many side effects (Persson et al., 2018).

Alternatively, intra-articular interventions are possible through injections of glycosaminoglycans (GAGs), in particular, hyaluronic acid (HA) (Nouri et al., 2022; Li et al., 2024). For several decades, HA has been exploited for its viscosupplementation effects, since it has been demonstrated that in OA joints, the composition of synovial fluid (fundamental for joint mechanics) is altered and hyaluronan is progressively degraded (Bhuanantanondh et al., 2011). Hence, HA intra-articular injections can improve synovial fluid rheological properties, thus promoting articular movement and reducing pain (Bhuanantanondh et al., 2011; Salgado et al., 2021). As regards viscoelasticity, HA is considered the main player; however, recent studies have reported that other proteoglycans and/or GAGs can contribute to reconstituting joint biochemical crosstalk (Lin et al., 2020).

Specifically, among the most studied macromolecules is chondroitin sulfate (CS); the latter is present in all vertebrates and invertebrates and plays a role in several biological processes (e.g., cell growth and anti-inflammatory activity) (Volpi, 2009; Stellavato et al., 2021). It is interesting to note that functional properties are closely related to different sulfation patterns and extractive sources (Volpi, 2019). Even more intriguing is the production of unsulfated chondroitin, namely, biofermentative chondroitin (BC), through a well-established fermentative process (D’Ambrosio et al., 2020). Previous human synoviocytes intra-proteome and secretome *in vitro* studies displayed an analogous trend between CS- and BC-treated cells, but a significant efficacy of BC’s anti-inflammatory capacity was observed (Russo et al., 2020; Vassallo et al., 2022). More recently, a hybrid complex, based on high molecular weight hyaluronan (HHA) and BC (HHA/BC), thus coupling HHA viscosupplementation properties and BC anti-inflammatory and degradative abilities, has been obtained, and its biochemical and biological features have been assayed both *in vitro* and *in vivo* (Vassallo et al., 2021; Papalia et al., 2021; Vassallo et al., 2023).

Another emerging OA biological therapy involves intra-articular injections of platelet-rich plasma (PRP) (Bennell et al., 2017). PRP is a fraction of plasma obtained by centrifugation and separation of whole blood. It represents an autologous source of high-level growth factors useful in various clinical applications (Weibrich et al., 2002; Crovetti et al., 2004; Akeda et al., 2006). Scientific studies have shown that PRP may affect chondrocyte-specific protein expressions (Spreafico et al., 2009). Specifically, PRP’s anti-inflammatory effect occurs through the inhibition of NF-κB activation and the enhancement of cellular proliferation and/or extracellular matrix (ECM) production by chondrocytes (Bendinelli et al., 2010; Spreafico et al., 2009; Akeda et al., 2006). Several clinical trials have compared the efficacy and safety of intra-articular PRP and HA in OA patients, but the results were highly controversial, and no clear advantages were demonstrated from using one or the other (Tang et al., 2020; Belk et al., 2021; Wu et al., 2020, Conza et al., 2024).

In this scientific context, the possibility of evaluating the bioactivity of these formulations on a robust *in vitro* cellular model is of great interest, also in light of the general interest in limiting animal-based studies. For this reason, the effects of HHA/BC combined with PRP were further investigated in this study, and the results presented here provide insights into the advantages in cartilage tissue repair. In fact, HA and BC restore the viscoelasticity of the synovial fluid and reduce the inflammatory process, respectively, while PRP improves chondrocyte anabolic activity with ECM component biosynthesis promoting cartilage tissue homeostasis. Therefore, the rheological and biological performances of a formulation containing HHA/BC and PRP were assayed and compared to HHA/BC alone. A comprehensive rheological characterization was performed under physiological conditions. The biological effects were evaluated through *in vitro* tests on human pathological chondrocytes and finally, a 3D-like *in vitro* culture was attempted to better mimic a cartilage/matrix structure and analyze the modulation of specific biomarkers after a longer period.

## 2 Materials and methods

### 2.1 Raw materials

Sinogel^®^ 3 mL (lot A0467, expiration 05/2025), also referred to as HHA/BC was kindly provided by IBSA Farmaceutici Italia (Lodi, Italy). It contains hyaluronic acid sodium salt 2.4% w/v and unsulfated chondroitin 1.6% w/v processed to obtain stabilized hybrid cooperative complexes, through the patented thermal treatment (Vassallo et al., 2023).

The platelet‐rich plasma (PRP) was obtained in lyophilized form by the research group of Prof. Ranieri Cancedda and Prof. Maddalena Mastrogiacomo of the University of Genoa, who took steps to collect and prepare the PRP according to the protocol patented in 2012EP2920297A1, producing a concentrate of 2.5 × 10^6^ cells/μL (Muraglia et al., 2014).

### 2.2 Rheological characterization

#### 2.2.1 Sample preparation

To prepare the formulations of HHA/BC+PRP, the following stock solutions were first made:

• Sol A) Lyophilic PRP was resuspended in 1 mL of sterile water to obtain a solution of 2.5 × 10^6^ cells/μL. This solution was diluted in Dulbecco’s phosphate buffered saline (PBS without calcium and magnesium, Thermo Fisher Scientific, Waltham, MA, United States of America) to reach the final concentration of 1.8 × 10^6^ cells/mL.
• Sol B) An appropriate amount of BSA powder (Sigma Aldrich, Milan, Italy) was dissolved in PBS to obtain a solution at 70 mg/mL.
• Sol C) An appropriate amount of BSA powder was dissolved in Sol A to obtain a 70 mg/mL solution in PRP and PBS.

These stock solutions were mixed to obtain the samples as follow:

a) HHA/BC 1:2 in PBS: an equal volume of Sinogel^®^ and PBS was mixed.
b) HHA/BC + PRP 1:2 in PBS: an equal volume of Sinogel^®^ and Sol A was mixed.
c) HHA/BC + BSA 7% 1:2 in PBS: an equal volume of Sinogel^®^ and Sol B was mixed.
d) HHA/BC + BSA 7% + PRP 1:2 in PBS: An equal volume of Sinogel^®^ and Sol C was mixed.

The samples were incubated overnight (15–16 h) at 37 °C with a stirring of 60 rpm.

#### 2.2.2 Rheological measurements

Samples rheological behaviour was studied using an oscillatory rheometer Physica MCR301 by Anton Paar (Ostfildern-Scharnhausen, Germany), equipped with a stainless-steel cone-plate measuring system (plate diameter 5 cm, cone angle 2°, truncation 0.207 µm) and a Peltier system for temperature regulation. Initially, an amplitude sweep test was conducted at a constant frequency of 1.59 Hz and a constant temperature of 37  °C in the 0.01%–100% strain range to delimit the linear viscoelasticity range (LVR) of all the formulations and three measurements were carried out for each sample.

Subsequently, the mechanical spectra of the formulations were derived as a function of the frequency in the range 0.159–15.9 Hz at a constant temperature of 37 °C and a constant strain of 2% (value belonging to the above linear viscoelasticity regime). For each measurement 21 points were acquired with 10 points per decade in the “no time setting” mode and at least three replicates were made for each sample.

Finally, rotational measurements were conducted to derive dynamic viscosity profiles; flow curves were obtained as a function of shear rate from 0.1 to 1,000 s^−1^ with a logarithmic ramp and 50 measuring points in “no time setting” mode. At least three measurements were executed for each sample at a constant temperature of 37 °C.

The rheological profiles of elastic and viscous modulus, tan δ and complex viscosity obtained either by variable amplitude testing or by frequency testing are given in the results section. In addition, dynamic viscosity flow curves derived under stationary conditions are reported.

### 2.3 Osteoarthritis *in vitro* model set up

OA *in vitro* model was obtained following the experimental protocols previously reported (Russo et al., 2020; Vassallo et al., 2021). Specifically, the Department of Medical and Surgical Specialties and Dentistry, University of Campania “Luigi Vanvitelli” (Naples, Italy) provided knee cartilage samples coming from patients undergoing total knee arthroplasty for end-stage osteoarthritis patients. All samples were classified as grade IV based on preoperative radiographic evaluation, according to the Kellgren-Lawrence grading system (Kellgren and Lawrence, 1957). The Internal Ethical Committee previously authorized all the experimental procedures (AOU-SUN reg. no. 0003711/2015). Once in the laboratory, cartilage tissues were washed with PBS, cut with sterile scalpel, and processed using an enzymatic digestion solution based on collagenase type I at 3 mg/mL and dispase at 4 mg/mL and left overnight at 37 °C on a shaking plate. After that, the obtained solution was filtered (70 μm, BD, Falcon, Franklin Lakes, NJ, United States) and centrifuged at 1,500 rpm for 10 min (Eppendorf, Hamburg, Germany). Thus, the cellular pellet was washed with PBS and re-centrifuged. Finally, the cells were re-suspended in Dulbecco’s modified eagle medium (DMEM) (Gibco, Carlsbad, CA, United States) supplemented with Fetal Bovine Serum (FBS) (Gibco, Carlsbad, CA, United States) (10% v/v), penicillin-streptomycin (Gibco, Carlsbad, CA, United States) (1% v/v), and Amphotericin B (Gibco, Carlsbad, CA, United States) (1% v/v). The primary chondrocytes were maintained at 37 °C in a humidified atmosphere with 5% v/v CO_2_ and the culture medium was changed every 48 h. Cellular phenotype was confirmed as previously reported by Stellavato and collaborators (La Gatta et al., 2021). For all the experiments, chondrocytes at 2^nd^ or 3^rd^ passage of *in vitro* culture were used.

### 2.4 GAG-based treatments on *in vitro* cell cultures

#### 2.4.1 2D *in vitro* culture

Primary chondrocytes were seeded in standard plates and incubated in the FBS-supplemented DMEM (untreated pathological cells, pCTR), or in the presence of HHA/BC or HHA/BC+PRP. Specifically, both HHA/BC and HHA/BC+PRP samples were prepared using the same culture medium described above for cell growth; HHA/BC was obtained by diluting Sinogel^®^ 1:4 w/v, while HHA/BC+PRP was prepared by diluting Sinogel^®^ 1:4 w/v and PRP 1:20 v/v in the culture medium.

#### 2.4.2 3D-like *in vitro* culture

A 3D-like chondrocytes culture was accomplished mixing around 20 × 10^4^ cells with 200 μL of HHA/BC, with or without 40 μL of PRP, and seeding the resulting suspension in a 12-well transwell with 0.4 μm pore size (Corning Incorporated Costar^®^, Corning, NY, United States). 20 μL of CaCl_2_ were quickly added to the formulations, while 600 μL of culture medium were supplemented below the transwell membranes. The cells were cultivated *in vitro* for 14 days, and the medium was changed every 48 h. Chondrocytes without any GAGs or PRP were seeded, on the top of the transwell as controls for comparative gene and protein expression analyses.

### 2.5 Biological evaluation

#### 2.5.1 Cellular viability assay

After 72 h of growth in 24-well standard-plate (25 × 10^3^ cells/well), Cell Counting Kit-8 (Dojindo EU GmbH, Munchen, Germany) was employed, following the manufacturer’s instructions, to assess cellular viability (Vassallo et al., 2023). Specific optical densities of each supernatant were measured at 450 nm using a Beckman DU 640 spectrometer (Beckman, Milan, Italy). The relative cell viability was calculated as a percentage of the maximal absorbance as reported in Equation 1:

$$Viability\;\left(\%\right)=\frac{mean\;\;OD\;\;treated\;cells}{mean\;\;OD\;\;untreated\;cells}\times 100$$ (1)

These outcomes were expressed as mean values of triplicates ±SD and two-tailed t-test was used to assess significative differences among HHA/BC and HHA/BC+PRP-chondrocytes samples and pCTR. p < 0.05 was considered statistically relevant.

#### 2.5.2 Gene expression analyses by quantitative Real Time-PCR

To assess the ability of HHA/BC alone and coupled to PRP in modulating specific OA-related gene expression, primary chondrocytes were seeded on a 24-well standard plate (30 × 10^3^ cells/well). After 24 h of treatments, the cells were harvested using TRIzol^®^ Reagent (Invitrogen, Milan, Italy), total intracellular RNA was isolated as reported by Vassallo et al. (2023) and reversely transcribed into cDNA following the manufacturer’s protocol (Reverse Transcription System Kit, Promega, Milan, Italy). Successively, for each sample, a quantitative Real Time-PCR (qRT-PCR) was performed by using the IQ™ SYBR^®^ Green Supermix (Bio-Rad Laboratories, Milan, Italy). The specific primer (Roche, Basel, Switzerland) sequences used in this experimental set-up are reported in Table 1. All the samples were analyzed in triplicate and glyceraldehyde-3-phosphate dehydrogenase (GAPDH) was here employed as housekeeping to normalize mRNA expression of genes. Eventual gene expression modulation with respect to pCTR was calculated by applying the Livak method (2^−ΔΔCt^) and using Bio-Rad iQ5 software (Bio-Rad Laboratories, Milan, Italy) (Vassallo et al., 2023). The same experimental procedure was employed for the cells grown on transwell (for 14 days). All data were presented as mean ± SD. These experiments were performed both in 2D and 3D-like *in vitro* cell cultures.

**TABLE 1:** Primer sequences used in qRT-PCR.

Gene name	PCR primer sequence 5’→ 3′
Glyceraldehyde-3-phosphate dehydrogenase (GAPDH)	TGC​ACC​ACC​AAC​TGC​TTA​GC GGC​ATG​GAC​TGT​GGT​CAT​GAG
Aggrecan (ACAN)	TCGAGGACAGCGAGGCC TCGAGGGTGTAGCGTGTAGAG
Type I collagen (COL1A1)	CCAGAAGAACTGGTACATCA CCGCCATACTCGAACTGGAA
Type II collagen (COL2A1)	CAACACTGCCAACGTCCAGAT CTGCTTCGTCCAGATAGGCAA
Matrix metallopeptidase 13 (MMP-13)	TCCCTGAAGGGAAGGAGC CTCGTCCAGGATGGCGTAG
Interleukin-6 (IL-6)	GTG​GAG​ATT​GTT​GCC​ATC​AAC​G CAG​TGG​ATG​CAG​GGA​TGA​TGT​TCT​G
Tumor necrosis factor alpha (TNF-α)	CGAGTGACAAGCCTGTAGC GGTGTGGGTGAGGAGCACAT

#### 2.5.3 Protein analyses by western blotting

Primary chondrocytes were seeded in a 12-well standard plate (BD, Falcon, Franklin Lakes, NJ, United States) (40 × 10^3^ cells/well) to perform Western blotting (WB) analyses as previously described (Vassallo et al., 2023). In detail, after 48 and 96 h, total intracellular proteins were extracted from each sample by a Radio-Immunoprecipitation Assay (RIPA buffer 1×; Cell Signaling Technology, Danvers, MA, United States) and quantified by protein assay reagent (Bio-Rad Laboratories, Milan, Italy). A total of 15 µg of protein was electrophoretically separated on a 10% SDS-PAGE polyacrylamide gel and transferred onto nitrocellulose membrane (GE, Amersham, United Kingdom). This latter was washed with tris-buffered saline containing 0.05% v/v Tween-20 (Bio-Rad Laboratories, Milan, Italy) (TTBS) and blocked with 5% w/v non-fat milk (Bio-Rad Laboratories, Milan, Italy) in TTBS for 30 min and successively incubated overnight at 4  °C with primary antibodies (all diluted 1:500 v/v) against COMP-2 (Santa Cruz Biotechnology, Dallas, TX, United States), NF-κB (Santa Cruz Biotechnology, Dallas, TX, United States), MMP-13 (Santa Cruz Biotechnology, Dallas, TX, United States), Collagen 2A1 (COL2A1) (Elabscience, Houston, TX, United States), Aggrecan (ACAN) (Santa Cruz Biotechnology, Dallas, TX, United States) and HAS-2 (Santa Cruz Biotechnology, Dallas, TX, United States). The following day, the membrane was gently washed with TTBS and incubated with specific secondary horseradish peroxidase-conjugated antibodies (Santa Cruz Biotechnology, Dallas, TX, United States) diluted 1:1,000 v/v for 2 h at room temperature. Blots were developed using an ECL detection system (GE, Amersham, United Kingdom). As a gel loading control, an actin antibody (Santa Cruz Biotechnology, Dallas, TX, United States) was used (diluted 1:1000 v/v) and a semi-quantitative protein expression analysis was performed using ImageJ software (National Institutes of Health campus, Bethesda, MD, United States).

#### 2.5.4 OA-related proteins secretion evaluation by bioplex assay

Quantitative analysis of specific OA-related biomarkers secretion by chondrocytes was performed through the Bioplex multiplex system (Bio-Rad Laboratories, Milan, Italy). Briefly, the conditioned media were collected after 48 h of chondrocytes treatment with HHA/BC and/or HHA/BC+PRP as previously described (Russo et al., 2020). According to manufacturer’s instructions, the concentration of eight analytes was simultaneously evaluated: interleukin of type 2, 4, 6, 8 and 10 (IL-2, IL-4, IL-6, IL-8, IL-10), Granulocyte-colony stimulating factor (G-CSF), Granulocyte macrophage colony stimulating factor (GM-CSF), Interferon (INF)γ, Tumor necrosis factor alpha (TNFα). A Bioplex MAGPIX Multiplex Reader system (Bio-Rad Laboratories, Milan, Italy) was used to acquire the data.

These experiments were performed both in 2D and 3D-like *in vitro* cell cultures.

#### 2.5.5 Immunofluorescence staining

COL2A1 and ACAN protein expressions were evaluated also by immunofluorescence staining (IF). Thus, the cells were seeded (5 × 10^3^ cells/well) in two chamber slides (BD, Falcon, Franklin Lakes, NJ, United States) and *in vitro* cultivated in the presence or absence of HHA/BC or HHA/BC+PRP for 72 h. Then, the supernatants were discarded, and the samples were washed twice with PBS and fixed with paraformaldehyde 4% w/v (Sigma Aldrich, Milan, Italy). In addition, the cells seeded on transwells were fixed with paraformaldehyde 4% w/v and the membranes removed from the plate. For all the samples, a solution containing Triton X-100 at 0.2% v/v (Sigma Aldrich, Milan, Italy) in PBS was employed to permeabilize the chondrocytes, after that, the primary antibodies against COL2A1 (Elabscience, Houston, TX, United States) (diluted 1:100 v/v) and ACAN (Elabscience, Houston, TX, United States) (diluted 1:100 v/v) were incubated with the samples overnight at 4 °C. Then, the slices were incubated with specific FITC-conjugated secondary antibodies (ThermoFisher Scientific, Waltham, MA, United States) (diluted 1:200) for 1 h at room temperature and covered using a mounting medium with DAPI-aqueous (Prodotti Gianni, Milan, Italy). The relative images were captured through an Axiovert 200 (Zeiss, Oberkochen, Germany) fluorescence microscope and analyzed using AxioVision 4.8.2. These experiments were performed both in 2D and 3D-like *in vitro* cell cultures.

#### 2.5.6 Wound healing on *in vitro* model of primary pathological chondrocytes

50 × 10^3^ cells were seeded in a 24-well standard plate (BD, Falcon, Franklin Lakes, NJ, United States) and incubated for 24 h at 37 °C and 5% CO_2_ v/v until 90%–95% confluence was reached. Scratch wounds were mechanically generated by a sterile pipette tip (Ø = 0.1 mm). Eventually detached cells and debris were washed away using PBS. Fresh culture medium supplemented with 2% v/v FBS and HHA/BC or HHA/BC+PRP was quickly added to chondrocytes. For the untreated cells, the same amount of GAG-based gel was replaced by PBS. The rate of wound closure was monitored by Time-Lapse Video-Microscopy (TLVM). Specific pictures of the scratched monolayers were captured every 60 min and analyzed using OKO Vision 4.3 software (Okolab, Naples, Italy).

### 2.6 Statistical analysis

For the rheological measurements, an ANOVA analysis with Tukey *post hoc* correction was performed to compare the tested samples in terms of G′, G″, tan δ and η* at 0.5 and 2.5 Hz, and zero-shear viscosity (η_0_). p values lower than 0.05 were considered for statistical differences.

For the biological experiments, a 2-tailed t-test was performed to compare the results obtained for the GAG-based treatments and the untreated chondrocytes (pCTR), and between HHA/BC and HHA/BC+PRP. p values below 0.05 were considered statistically relevant.

## 3 Results

### 3.1 Rheological description

**FIGURE 1:**
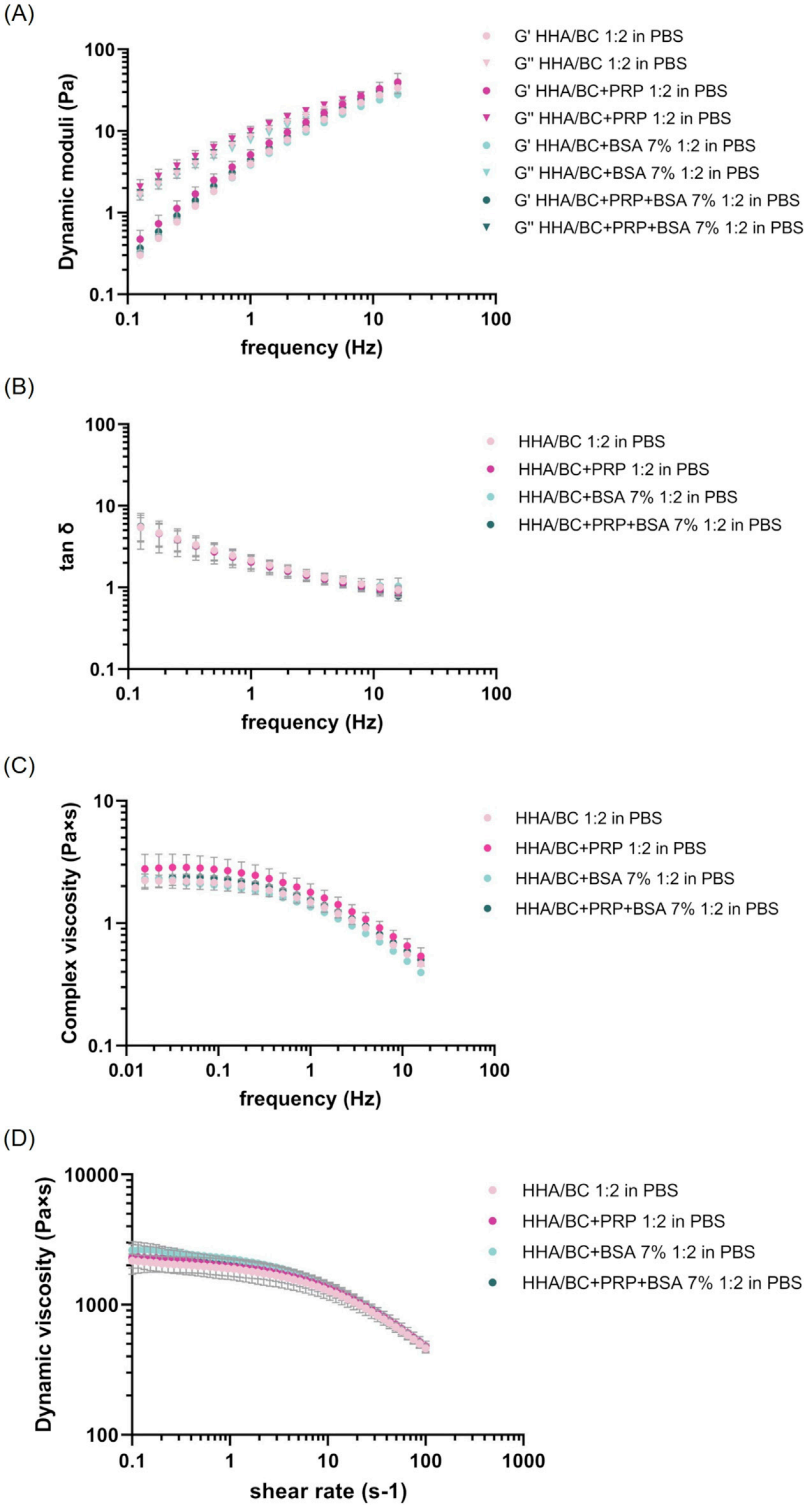
Rheological characterization of the formulations at 37 °C. **(A–C)** Mechanical spectra as a function of frequency from 0.159 to 15.9 Hz at 2% strain: **(A)** Storage and loss moduli; **(B)** tan δ; **(C)** complex viscosity. **(D)** Dynamic viscosity as a function of shear rate from 0.1 to 100 s^−1^.

Rheological results obtained from rotational and oscillatory measurements are reported in Figure 1.

The amplitude sweep test (Supplementary Figure S1), performed at a constant frequency and variable strain, highlighted the presence of a linear viscoelastic regime, in which both the storage modulus (Supplementary Figure S1A) and the loss modulus (Supplementary Figure S1B) kept a constant profile as deformation increased. A strain percentage of two was clearly within this range and was chosen as a constant value for frequency sweep tests.

The mechanical spectra (Figure 1A), tan delta (Figure 1B) and complex viscosity (Figure 1C) were obtained at a constant deformation and variable frequency within the range of clinical interest.

From the graph, the typical viscoelastic behaviour of low concentration hyaluronan-based formulations was distinguishable, with the viscous modulus prevailing over the elastic modulus at low frequency values and, conversely, the elastic modulus prevailing over the viscous one at high frequencies. The crossover point was detectable in all samples regardless of the presence of PRP or BSA at high frequency values. No significant differences were observed among samples with different PRP and BSA compositions compared to HHA/BC aqueous solution either in the strain test nor in the frequency sweep test. In this regard, Table 2 reports the values of the viscoelastic moduli, tan δ and complex viscosity extracted at 0.5 Hz and 2.5 Hz (frequency values corresponding to the knee cartilage behaviour during walking and running, respectively) expressed as mean ± SD.

**TABLE 2:** Dynamic moduli, tan δ and complex viscosity at 0.5 Hz and 2.5 Hz (walk frequency and run frequency in the knee cartilage).

Sample	G’ (Pa)	G’’ (Pa)	tan δ	η* (Pa × s)
0.5 Hz				
HHA/BC 1:2 in PBS	1.8 ± 0.3	5.1 ± 0.5	2.8 ± 0.3	1.7 ± 0.2
HHA/BC + PRP 1:2 in PBS	2.5 ± 1.1	6.3 ± 1.1	2.7 ± 0.8	2.1 ± 0.4
HHA/BC + BSA 7% 1:2 in PBS	1.8 ± 0.8	4.7 ± 0.9	2.8 ± 0.6	1.6 ± 0.4
HHA/BC + BSA 7% + PRP 1:2 in PBS	2.1 ± 0.9	5.4 ± 1.1	2.7 ± 0.6	1.8 ± 0.4
2.5 Hz				
HHA/BC 1:2 in PBS	10.6 ± 1.3	15.5 ± 1.6	1.5 ± 0.1	1.0 ± 0.1
HHA/BC + PRP 1:2 in PBS	12.8 ± 3.6	13.8 ± 1.4	1.4 ± 0.2	1.2 ± 0.2
HHA/BC + BSA 7% 1:2 in PBS	9.8 ± 2.5	17.7 ± 1.8	1.4 ± 0.2	1.0 ± 0.1
HHA/BC + BSA 7% + PRP 1:2 in PBS	11.3 ± 2.7	15.5 ± 0.7	1.4 ± 0.2	1.0 ± 0.2

Statistical analysis performed using ANOVA with Tukey *post hoc* correction allowed multiple comparisons of the samples and revealed p values > 0.05.

Moreover, Figure 1D shows the dynamic viscosity profile vs*.* shear rate for each sample tested.

Consistently with the previous set of measurements, the rheological behaviour of the HHA/BC solution under steady-state conditions was not influenced by the addition of platelet-rich-plasma or albumin. All samples, in fact, exhibited the shear thinning behaviour of pseudo-plastic fluids (as observed in linear high molecular weight hyaluronans), with the dynamic viscosity remaining constant at low shear rates and beginning to decrease as shear rate increased. The zero-shear-viscosity values, extracted within the Newtonian plateau viscosity range, are reported in Table 3 and were comparable among samples, also at statistical level. ANOVA analysis with Tukey *post hoc* correction revealed p > 0.05 for all sample pairs considered.

**TABLE 3:** Values of zero-shear viscosity extracted from the flow curves measured at 37 °C.

Sample	η_0_ (Pa × s)
HHA/BC 1:2 in PBS	2.1 ± 0.2
HHA/BC + PRP 1:2 in PBS	2.3 ± 0.4
HHA/BC + BSA 7% 1:2 in PBS	2.6 ± 0.3
HHA/BC + BSA 7% + PRP 1:2 in PBS	2.3 ± 0.1

### 3.2 Biological evaluation in 2D and 3D-like *in vitro* culture

#### 3.2.1 Cellular viability assay

**FIGURE 2:**
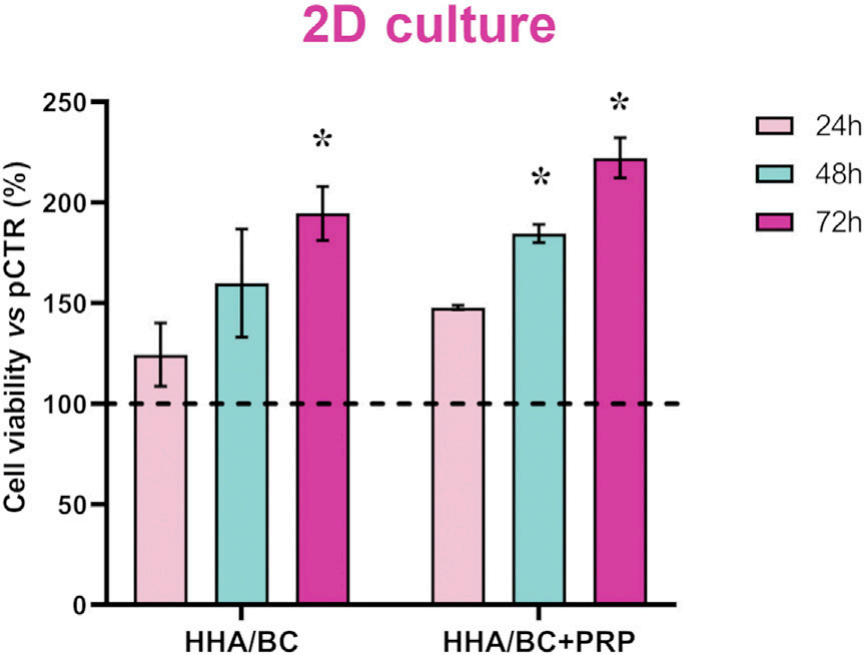
Cell viability assay performed using CCK-8 staining. Data are expressed as mean ± SD. Statistical analysis was performed using a t-test: *p < 0.05 vs. pCTR.

As expected, CCK-8 staining data showed that HHA/BC sustained chondrocytes viability and promoted their growth compared to untreated cells. However, as shown in Figure 2, the addition of PRP to HHA/BC had positive effects on cell proliferation. In fact, after 24 h, both treatments better sustained cell viability compared to pCTR, but HHA/BC+PRP chondrocytes also showed significantly (p < 0.05) higher viability than HHA/BC alone at 48 h, improving chondrocytes growth/viability by approximately 82% with respect to pCTR. Moreover, after 72 h of treatments, cell viability in the presence of HHA/BC+PRP and HHA/BC compared to untreated cells increased by approximately 122% and 94%, respectively.

#### 3.2.2 Gene expression analyses by qRT-PCR

**FIGURE 3:**
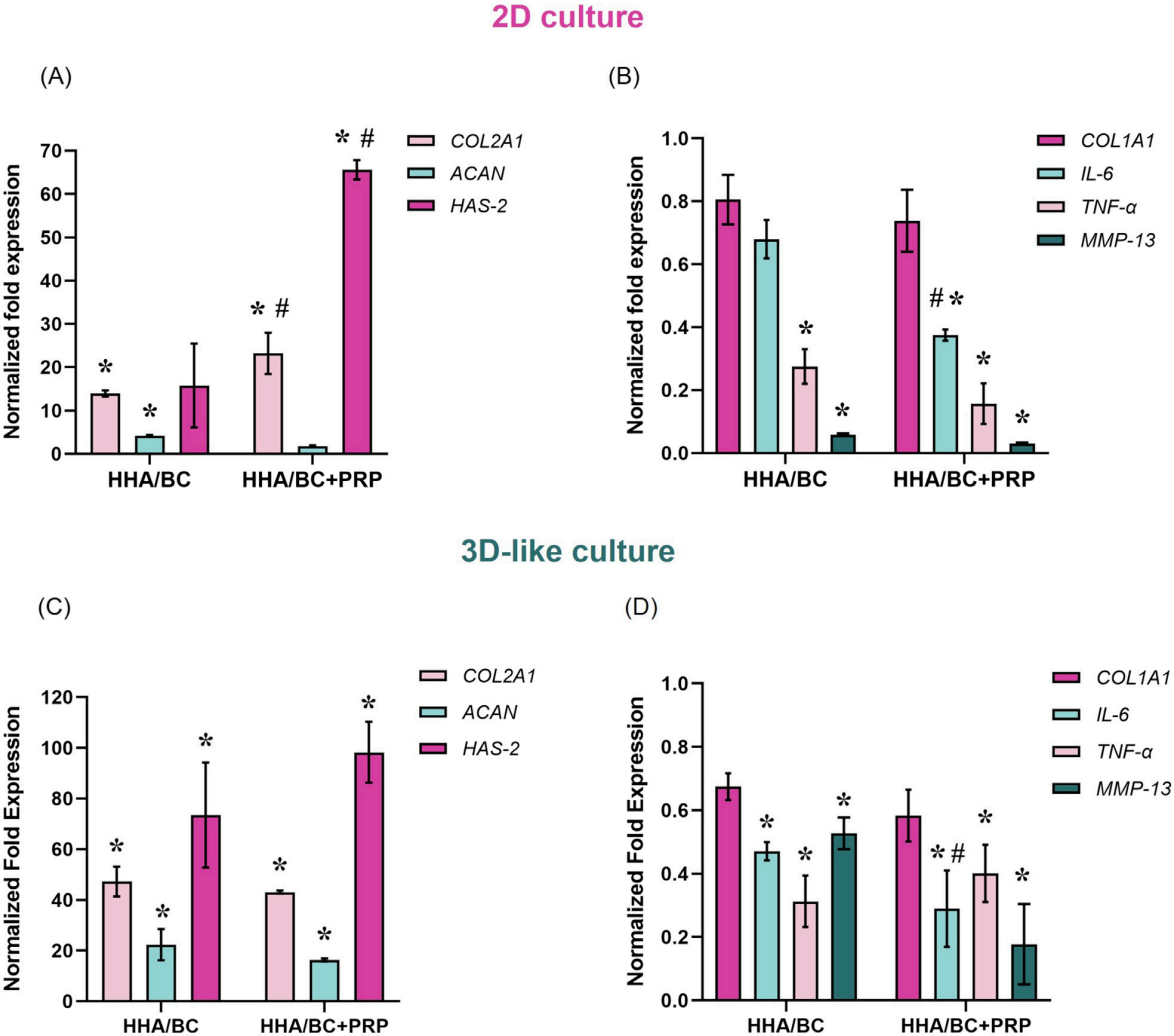
Gene expression analysis by qRT-PCR in 2D and 3D-like *in vitro* cultures. **(A)** *COL2A1*, *ACAN*, and *HAS-2* expression in 2D culture; **(B)** *COL1A1*, *IL-6*, *TNF-α*, and *MMP-13* expression in 2D culture; **(C)** *COL2A1*, *ACAN*, and *HAS-2* expression in 3D-like culture; **(D)** *COL1A1*, *IL-6*, *TNF-α*, and *MMP-13* expression in 3D-like culture. Data were normalized to pathological untreated cells (pCTR). Results are expressed as mean ± SD. Statistical analysis was performed using a t-test: *p < 0.05 vs*.* pCTR; #p < 0.05 for HHA/BC+PRP vs*.* HHA/BC.

The results of qRT-PCR experiments carried out in 2D, and 3D-like *in vitro* cultures are reported in Figure 3.

Specifically, qRT-PCR outcomes from 2D experiments revealed that both HHA/BC and HHA/BC+PRP significantly (p < 0.05) increased *COL2A1* (a specific biomarker of chondrocyte phenotype) and reduced *COL1A1* (a specific biomarker of fibroblast phenotype) with respect to pCTR. In fact, as shown in Figure 3A, *COL2A1* gene expression was upregulated in the presence of HHA/BC and HHA/BC+PRP by approximately 14- and 23-fold compared to pCTR, respectively. In this case, the efficacy of HHA/BC+PRP was significantly (p < 0.05) greater than that of HHA/BC. However, HHA/BC was more effective than HHA/BC+PRP in enhancing *ACAN* gene expression, a key component of the ECM, with a significant (p < 0.05) upregulation of approximately 4-fold with respect to pCTR. Furthermore, *HAS*-2 upregulation was significant (p < 0.05) in the presence of HHA/BC+PRP compared to untreated cells and HHA/BC. However, both samples succeeded in downregulating specific genes related to inflammation and cartilage degradation processes compared to pCTR (Figure 3B). In this context, HHA/BC+PRP was significantly (p < 0.05) more effective than HHA/BC in reducing *IL-6* expression level. Interestingly, for *MMP-13* and *TNF-α* downregulation, the treatments displayed a similar behaviour.

Gene expression analyses performed in a 3D-like *in vitro* culture confirmed and corroborated the results obtained in 2D experiments. In detail, *COL2A1* gene expression was significantly (p < 0.05) upregulated in the presence of HHA/BC and HHA/BC+PRP by approximately 47- and 43-fold compared to pCTR, respectively (Figure 3C). However, unlike the 2D *in vitro* culture, HHA/BC was slightly more efficient than HHA/BC+PRP. Similar results were obtained for *ACAN* gene expression with a significant (p < 0.05) increase compared to untreated cells of 22.3-fold in the presence of HHA/BC and 16.2-fold with HHA/BC+PRP. On the other hand, a similar and significant (p < 0.05) *HAS-2* upregulation relative to pCTR was found for both treatments. Moreover, data in Figure 3D showed that HHA/BC and HHA/BC+PRP similarly reduced *COL1A1* gene expression compared to untreated cells, confirming their efficacy. The *COL2A1*/*COL1A1* gene expression ratio was similar between the two treatments (73.4 ± 8.9 for HHA/BC and 74.7 ± 11.4 for HHA/BC+PRP). Finally, both formulations confirmed their ability to significantly (p < 0.05) decrease *IL-6, TNF-α* and *MMP-13* gene expression vs*.* pCTR. The reduction in *IL-6* expression was significantly different (p < 0.05) between HHA/BC and HHA/BC+PRP.

#### 3.2.3 Protein analyses by western blotting

**FIGURE 4:**
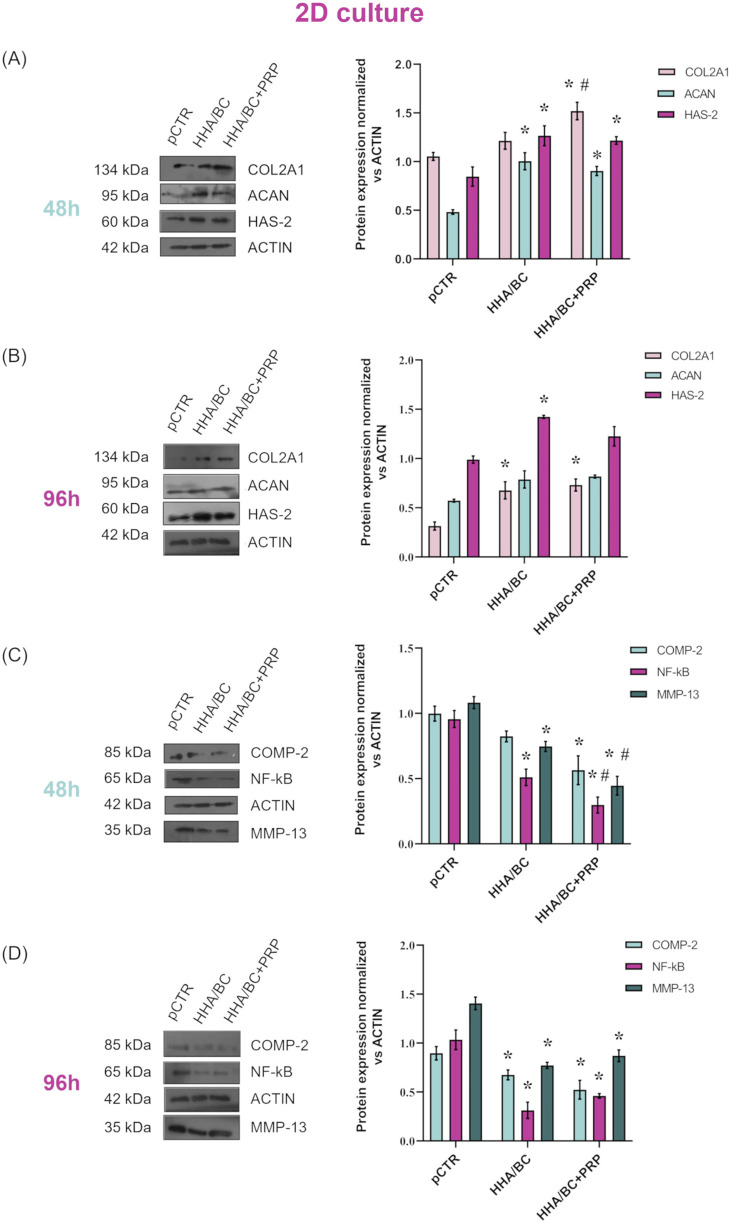
Protein expression levels evaluation by WB of COL2A1, ACAN and HAS-2 after 48 h **(A)** and 96 h **(B)** of GAG-based treatments. Protein expression levels evaluation by WB of COMP-2, NF-κB and MMP-13 after 48 h **(C)** and 96 h **(D)** of GAG-based treatments. Densitometric analyses were performed by normalizing each protein expression vs*.* ACTIN. Data are expressed as mean ± SD. Statistical analysis was performed using a t-test: *p < 0.05 vs*.* pCTR; #p < 0.05 for HHA/BC+PRP vs*.* HHA/BC.

Results of the Western blotting experiments are reported in Figure 4. Specifically, to assess the ability of the samples to maintain the chondrocyte phenotype and increase the production of specific cartilage components, COL2A1, ACAN, and HAS-2 protein expression modulations were evaluated and are presented here (Figures 4A,B). Figure 4A shows that both formulations enhanced the production of these biomarkers with respect to pCTR after 48 h. In fact, COL2A1 protein upregulation observed in HHA/BC+PRP-treated cells was significant (p < 0.05) compared to both pCTR and HHA/BC-treated cells, with an increase of approximately 1.51- and 1.21-fold, respectively. ACAN protein levels were significantly (p < 0.05) higher in the presence of both HHA/BC and HHA/BC+PRP (2- and 1.80-fold, respectively) with respect to untreated cells. HAS-2 protein expression was significantly (p < 0.05) increased by both samples (1.50- and 1.44-fold vs*.* pCTR with HHA/BC and HHA/BC+PRP, respectively). Figure 4B shows the modulation of protein expression of the same biomarkers after 96 h of treatment. It is interesting to note that baseline expression level of COL2A1 in pCTR was lower than that at 48 h. Also in this case, HHA/BC and HH/BC+PRP significantly (p < 0.05) upregulated COL2A1 level compared to untreated cells (2.10- and 2.30-fold, respectively). ACAN expression, compared to pCTR, was similarly sustained by both GAG-based formulations, with no significant differences. HAS-2 protein expression was significantly (p < 0.05) upregulated in the presence of HHA/BC.

The protein expression of the biomarkers related to inflammation and cartilage degradation processes, evaluated after 48 and 96 h, is presented in Figures 4C,D, respectively. As expected, untreated cells exhibited high protein levels of COMP-2, NF-κB, and MMP-13, confirming an ongoing inflammatory and cartilage degradation process. After 48 h, both tested formulations reduced the protein expression of these biomarkers compared to pCTR. Specifically, COMP-2 level decreased by 1.21-fold in the presence of HHA/BC and 1.76-fold with HHA/BC+PRP. Finally, HHA/BC+PRP was significantly (p < 0.05) more effective than HHA/BC in reducing NF-κB and MMP-13 protein expression relative to pCTR. In fact, NF-κB downregulation vs*.* pCTR was 1.88- and 3.27-fold with HHA/BC and HHA/BC+PRP, respectively. On the other hand, HHA/BC+PRP reduced MMP-13 protein expression compared to pCTR by approximately 2.45-fold, while in the presence of HHA/BC, this reduction was about 1.50-fold. Figure 4D shows that the formulations were significantly (p < 0.05) effective in reducing COMP-2 protein level vs*.* pCTR even after 96 h (1.38- and 1.63-fold in the presence of HHA/BC and HHA/BC+PRP, respectively). Interestingly, at this experimental time point, HHA/BC was more effective than HHA/BC+PRP in NF-κB and MMP-13 protein downregulation (approximately 4.16- and 2.77-fold reduction vs*.* pCTR for NF-κB; 1.93- and 1.70-fold decrease vs*.* pCTR for MMP-13).

#### 3.2.4 OA-related proteins secretion evaluation by bioplex assay

**FIGURE 5:**
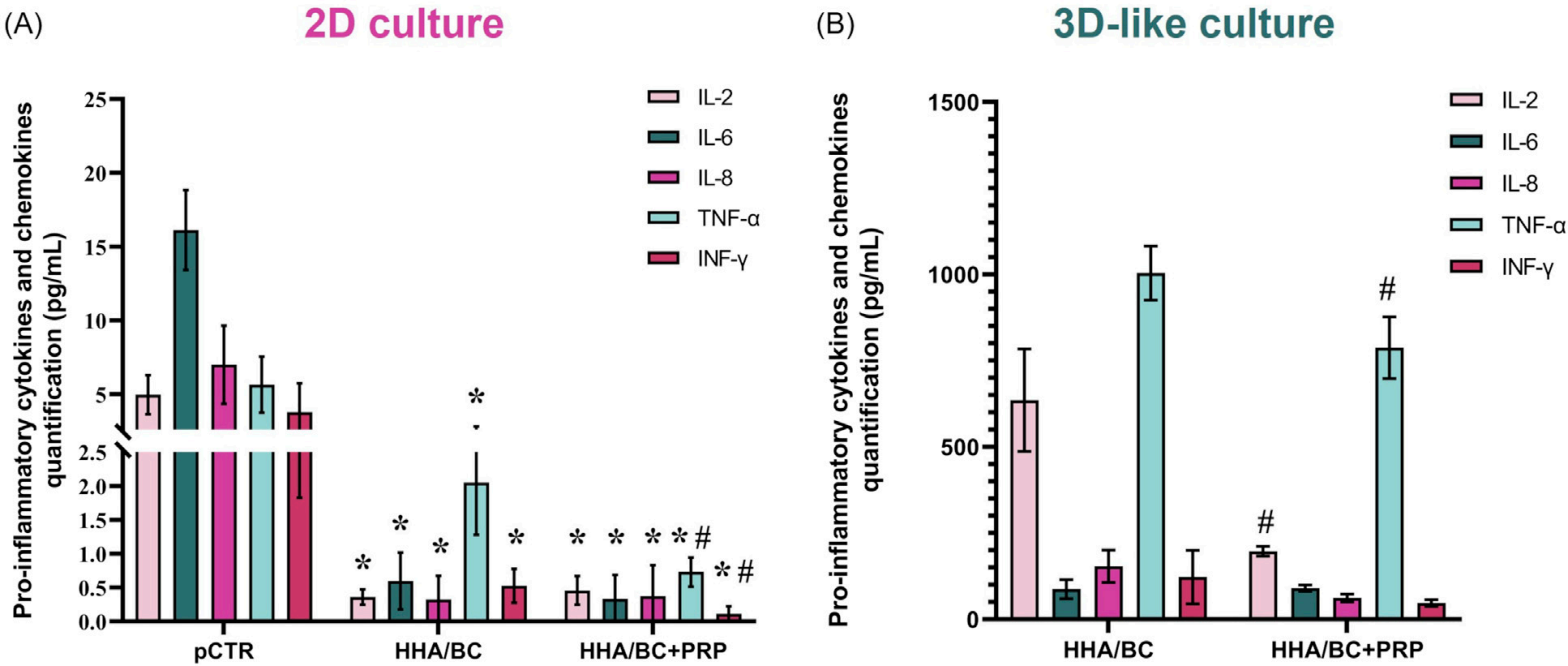
Bioplex assay in 2D **(A)** and 3D-like **(B)** *in vitro* culture. Evaluation of OA-related pro-inflammatory cytokines and chemokines in supernatants (pg/mL) of pathological chondrocytes treated for 48 h and 14 days with HHA/BC or HHA/BC+PRP. Data are expressed as mean ± SD. Statistical analysis was performed using a t-test: *p < 0.05 vs*.* pCTR; #p < 0.05 for HHA/BC+PRP vs*.* HHA/BC.

A Bioplex assay was performed to evaluate how the samples affected the secretion of specific biological mediators from pathological chondrocytes, both in 2D (Figure 5A) and 3D-like (Figure 5B) culture conditions.

Figure 5A shows that both HHA/BC and HHA/BC+PRP significantly (p < 0.05) reduced the secretion of the selected biomarkers vs*.* pCTR. Specifically, IL-2 protein secretion decreased by approximately 15- and 11-fold compared to untreated cells in the presence of HHA/BC and HHA/BC+PRP, respectively. Similarly, IL-6 protein level in cell supernatants was reduced by 27.3- and 48.8-fold vs*.* pCTR with HHA/BC and HHA/BC+PRP, respectively. Moreover, HHA/BC+PRP downregulated IL-8 protein secretion by approximately 10.4-fold, while HHA/BC decreased it by 23.3-fold vs*.* pCTR. Finally, HHA/BC+PRP significantly (p < 0.05) affected TNF-α and INF-γ protein levels compared to both pCTR and HHA/BC. Specifically, TNF-α secretion was reduced by 2.75- and 7.73-fold compared to pCTR in the presence of HHA/BC and HHA/BC+PRP, respectively. On the other hand, INF-γ levels decreased vs*.* pCTR with HHA/BC by 5.34-fold, while with HHA/BC+PRP this reduction was about 25.3-fold.

Figure 5B displays that the addition of PRP to HHA/BC improved its ability to modulate the analytes considered. In detail, IL-2 protein secretion decreased by approximately 3-fold in HHA/BC+PRP (p < 0.05) compared to HHA/BC. The reduction of IL-8 and INF-γ obtained in HHA/BC+PRP vs*.* HHA/BC was very similar (about 2.50-fold). Finally, TNF-α was significantly lower in HHA/BC+PRP (p < 0.05) with respect to HHA/BC, while the IL-6 values were similar between the samples.

#### 3.2.5 Immunofluorescence staining

**FIGURE 6:**
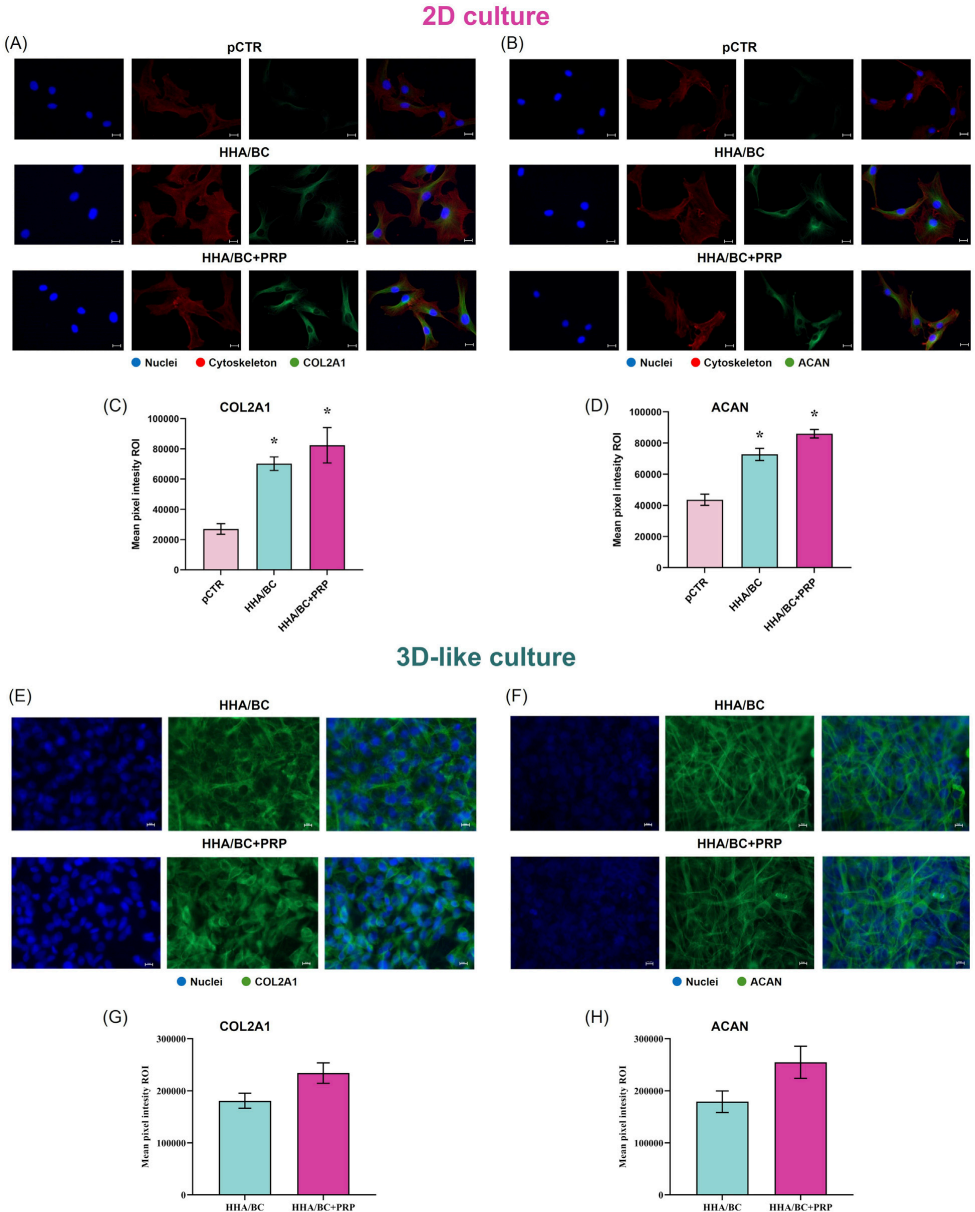
IF staining and quantification of COL2A1 and ACAN in 2D and 3D-like *in vitro* culture. **(A)** IF images of COL2A1 in 2D culture; **(B)** IF images of ACAN in 2D culture; **(C)** quantification of COL2A1 mean pixel fluorescence intensity in 2D culture; **(D)** quantification of ACAN mean pixel fluorescence intensity in 2D culture; **(E)** IF images of COL2A1 in 3D-like culture; **(F)** IF images of ACAN in 3D-like culture; **(G)** quantification of COL2A1 mean pixel fluorescence intensity in 3D-like culture; **(H)** quantification of ACAN mean pixel fluorescence intensity in 3D-like culture. Representative micrographs: FITC green antibody was used for collagen and aggrecan detection, while nuclei were stained in blue and cytoskeleton in red. Scale bar 10 μm, objective magnification ×40. Data are presented as mean ± SD. Statistical analysis was performed using a t-test: *p < 0.05 vs*.* pCTR; #p < 0.05 for HHA/BC+PRP vs*.* HHA/BC.

Immunofluorescence staining results from 2D and 3D-like culture conditions are reported in Figure 6. The images obtained from 2D experiments (Figures 6A,B) show that both COL2A1 and ACAN green fluorescence signals were less intense in pCTR compared to treated cells. Specifically, the results (Figures 6C,D) highlighted that HHA/BC and HHA/BC+PRP strongly increased COL2A1 protein expression vs*.* pCTR (p < 0.05), thus preserving chondrocyte-specific features. Similarly, a higher intensity signal was found for ACAN in the presence of the two treatments compared to pCTR (p < 0.05). The comparison of the two formulations revealed that HHA/BC+PRP was slightly more efficient than HHA/BC in enhancing ACAN and COL2A1 protein expression. Figures 6E,F display the immunofluorescence staining performed for COL2A1 and ACAN, respectively, on pathological chondrocytes grown for 2 weeks in a transwell insert in the presence of HHA/BC or HHA/BC+PRP. The results (Figures 6G,H) showed that the number of cells increased after 48 h of treatment, confirming the biocompatibility of these formulations and their ability to support cell growth. Furthermore, in both treatments, a similar protein expression of COL2A1 and ACAN was found.

#### 3.2.6 *In vitro* scratch assay

**FIGURE 7:**
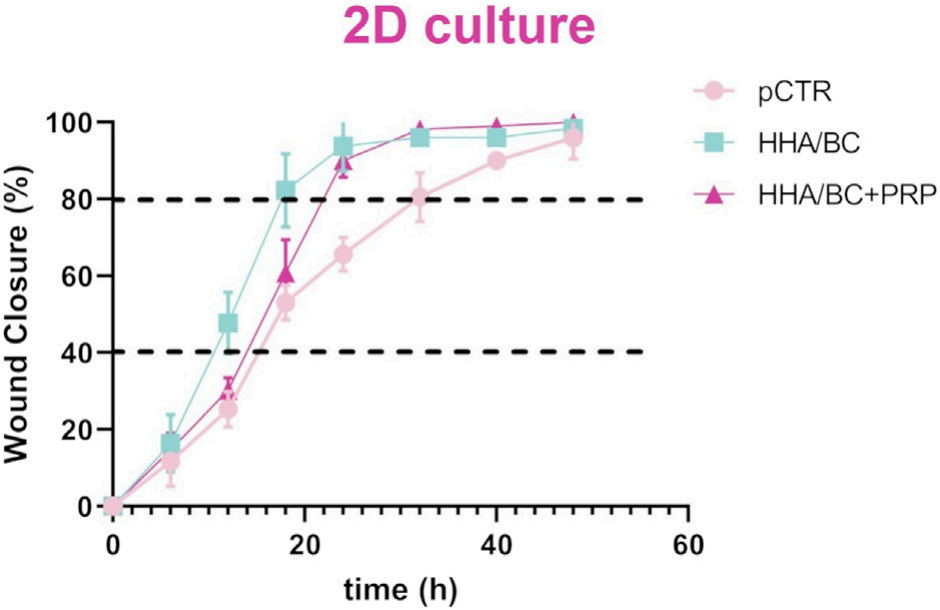
Repair area (%) for the untreated cells and the cells treated with HHA/BC and HHA/BC+PRP. The curves are the results of the mean values of four different captured fields of view ±SD.

Figure 7 shows that the scratch in pathological chondrocytes healed faster in the presence of both HHA/BC and HHA/BC+PRP compared to pCTR. In fact, after 32 h with the treatments, cells achieved 98% wound closure, while the untreated chondrocytes reached the same repair target within 50 h. Wound closure appeared similar in the first 6 h; however, image analysis and evaluation of the remaining scratched area revealed a faster closure for HHA/BC during the first 12 h compared to HHA/BC+PRP and untreated chondrocytes. After that, chondrocyte migration speed became comparable between the two formulations. After 24 h of incubation, no relevant differences were observed between HHA/BC and HHA/BC+PRP, while the closure rate achieved (≃ 92%) by both the samples within 24 h was significantly higher than pCTR (p < 0.05). For clarity, the incubation times required by all samples to reach 40% and 80% wound closure were also derived and reported as mean ± SD. pCTR showed an average time of 15.0 ± 5.6 h to reach 40% wound closure and 35.8 ± 6.5 h to reach 80%. The sample treated with HHA/BC demonstrated a significantly faster wound healing, achieving 40% closure in 10.9 ± 0.5 h and 80% in 18.2 ± 1.8 h. Lastly, the HHA/BC+PRP sample reached 40% closure in 14.7 ± 0.6 h and 80% in 20.9 ± 1.4 h.

## 4 Discussion

Viscosupplementation through intra-articular injection of glycosaminoglycans is one of the most widely used treatments for osteoarthritis (Bhuanantanondh et al., 2011; Salgado et al., 2021; Bernstein, 2004). Several intra-articular injectables contain not only hyaluronic acid but also other GAGs, primarily chondroitin sulfate (Volpi, 2009; Volpi, 2019). Recent studies have confirmed that CS, when combined with other GAGs (e.g., HA) enhances chondrocyte viability and reduces the activation of pro-inflammatory mediators (Terencio et al., 2016; Simental-Mendía et al., 2018). However, ethical and religious concerns related to the use of animal-derived products have driven scientific interest toward the development of alternative sources. In this context, Vessella et al. (2020) and Volpi and Maccari (2003) recently explored the use of sulfated chondroitin obtained through a semisynthetic approach.

As previously described, the biological effects of biofermentative unsulfated chondroitin compared to CS have been extensively studied in several OA *in vitro* models (Russo et al., 2020; Vassallo et al., 2022). Furthermore, the combination of high molecular weight hyaluronan with BC has been thoroughly characterized in terms of its viscoelastic properties and resistance to enzymatic degradation. More recently, its beneficial effects on pro-inflammatory biomarkers in human pathological chondrocytes have also been demonstrated (Vassallo et al., 2021; Vassallo et al., 2023). According to Vassallo et al. (2023), the HHA/BC formulation exhibited rheological properties that support its potential to manage mechanical stress in the synovial joints during walking and running, thereby providing effective viscosupplementation benefits.

Additionally, platelet-rich plasma has gained attention in OA treatment due to its low cost and safety, as it is an autologous biological material with minimal risk of immunogenic reaction (Akeda et al., 2006). In fact, PRP is obtained from patient’s blood and injected into a damaged joint space or an area of surgical intervention (Crowley and Soti, 2023). PRP contains a high concentration of biological mediators (e.g., growth factors, cytokines), which play a key role in cartilage repair and regeneration by activating specific biochemical pathways in chondrocytes (Tang et al., 2020). However, PRP lacks significant mechanical properties, which has led to its recent combination with HA. These formulations have proven effective in counteracting cartilage degradation and promoting ECM regeneration (Chen et al., 2014). Unfortunately, the results of the available studies are inconsistent, and it is not clear whether therapy based on PRP use is more effective than that with HA (Crowley and Soti, 2023).

Russo and collaborators (Russo et al., 2016) investigated the rheological and biological properties of various HA-based formulations for OA treatment, including a hybrid complex of high and low molecular weight HA (HL) combined with PRP. Their study found no significant differences in cell viability between HA formulations with or without PRP; however, HL improved chondrocyte anabolic activity. Similarly, Chen and collaborators (Chen et al., 2014) confirmed that combining HA of different molecular weights with PRP reduced inflammation-related pathways in chondrocytes. These findings suggest that the low molecular weight HA fraction may be responsible for the synergistic effect between HA and PRP. Based on these data, the present study aimed to investigate the rheological properties and biological effects of HHA/BC combined with PRP in primary human OA chondrocytes. Given its molecular weight of approximately 35–40 kDa, BC may play a crucial role in the synergistic interaction between GAGs and PRP.

The ability of the HHA/BC+PRP formulation to act as a viscosupplement, protecting, replenishing, and replacing synovial fluid lost due to joint cartilage damage, was assessed through rheological analysis in the presence or absence of bovine serum albumin. The viscoelasticity and the mechanical stress absorption capacity of these new formulations were evaluated and compared with well-established hybrid cooperative complex-based solutions. Rheological measurements, performed under both oscillatory and rotational conditions, demonstrated that the addition of PRP and/or BSA to the GAG-based formulations did not alter the typical rheological profile of linear polymers. Neither the qualitative mechanical spectra and flow curves nor the dynamic moduli at fixed frequencies and the zero-shear viscosity were affected. Furthermore, the behavior of the formulations in the presence of albumin was comparable to that of the other samples, indicating no significant interactions between proteins and glycosaminoglycans complex were occurring. Since the addition of albumin was intended to mimic the physiological protein content of synovial fluid, these results are relevant in view of the *in vivo* application; in these conditions HHA/BC will be able to positively influence the overall rheological performance as expected, also in the presence of PRP.

These findings are consistent with previous studies (Russo et al., 2016) on high molecular weight HA products (Sinovial^®^ 0.8% w/v, Sinovial^®^ 1.6% w/v and Hyalubrix^®^ 1.5% w/v) and high/low molecular weight HA hybrid complex (HHA/LHA)-based product (Sinovial^®^ HL 3.2%). They also support the compatibility of intra-articular injections combining not only HHA/LHA with PRP, but also the new high GAG concentration formula based on HHA/BC and PRP.

In agreement with Russo and collaborators (Russo et al., 2016), biological tests on human OA chondrocytes confirmed that the addition of PRP to GAG-based formulations significantly improved cell viability. To further explore the affected cellular pathways, additional analyses were conducted. Specifically, key biomarkers associated with OA progression (NF-κB, IL-6 and TNF-α) (Vincent, 2019) were evaluated at both the gene and protein levels in untreated and treated chondrocytes.

It is well-known that healthy cartilage maintains a balance between anabolic and catabolic processes in chondrocytes. However, under pathological conditions, catabolic processes predominate, leading to increased metalloprotease activity and decreased ECM synthesis. This imbalance also affects the chondrocyte phenotype under stress conditions (Vela-Anero et al., 2017).

In our 2D assays, NF-κB-related pro-inflammatory biomarkers were, as expected, highly active in pathological cells. However, treatment with both HHA/BC and HHA/BC+PRP significantly reduced NF-κB expression, as well as IL-6 and TNF-α levels, with PRP further enhancing the anti-inflammatory effects of HHA/BC alone. Remarkably, catabolic mediators such as MMP-13 and cartilage oligomeric matrix protein (COMP-2) were downregulated by treatments and more effectively by the PRP-containing formulation.

Another key aspect related to *in vitro* studies is prolonging cultivation to obtain an adequate number of cells, but this often leads, within a few passages of the primary cells, to a rapid de-differentiation with consequent loss of the specific cellular phenotype. Therefore, it is fundamental, at the same time, to maintain the specific phenotype, whilst promoting cell proliferation in order to be able to regenerate the cartilage (Amr et al., 2022). For this reason, markers related to chondrocyte phenotype maintenance (COL2A1), inhibition of metalloprotease activity (TIMP-2), HA biosynthesis (HAS-2) and ECM support (ACAN) were analyzed. Again, the PRP-containing formulation exhibited superior effects in improving the biochemical profile of pathological chondrocytes. Interestingly, human pathological chondrocytes embedded in the proposed gels, in 3D-like *in vitro* culture, confirmed the possibility to maintain the cell phenotype, furthermore HHA/BC+PRP enhanced the biosynthesis of ECM components such as COL2A1 and ACAN even better than HHA/BC alone. The pro-inflammatory cytokines released in the 3D cultures of the pathological chondrocytes embedded in the two gels here studied showed a similar profile to the 2D results. As rational, due to the prolonged incubation and higher cell density, the biomarkers concentration in 3D assays was higher, although chondrocytes grown in HHA/BC+PRP secreted lower inflammation-related analytes. Overall 3D-like cultures here proposed improved cell interactions showing a restored physiological metabolic activity of the chondrocytes, thus supporting the potential for cartilage regeneration of the hybrid complexes containing unsulfated biofermentative chondroitin also added with platelet rich plasma. Although, it is well-known that *in vitro* models do not fully replicate the complexity of the joint environment, lacking mechanical cues, immune interactions, and systemic influences, they still provide valuable insights into cellular mechanisms and serve as effective tools for the early-stage evaluation of therapeutic strategies (Salgado et al., 2021; Bartolotti et al., 2021). Therefore, the beneficial effects observed in these models may prove useful for clinical application, not only in the management of degenerative joint diseases but also in treating sport-related injuries to support faster recovery in athletes (Buckwalter, 2002).

## 5 Conclusion

In the framework of this study the formulations based on stabilized hybrid complexes made of high molecular weight hyaluronic acid and unsulfated chondroitin obtained by fermentative processes, were fully characterized by rheological measurements, in presence of proteins and PRP. Results assessed the possibility of using a combination of HHA/BC with PRP without affecting the viscoelasticity that is very important for intra-articular treatments of OA. Further experiments on OA *in vitro* model, also permitted to evaluate beneficial effects of the proposed treatments, with a specific improvement in the inflammation cascade. Remarkably it has been demonstrated that the formulations prompted the physiological biosynthesis of cartilage extracellular matrix components. The human primary chondrocytes were analyzed also in 3D cultures with remarkable positive outcomes. Overall, these results may support a beneficial effect in damaged joints cartilage by the injective treatments of HHA/BC alone and in combination with PRP, supporting clinicians in the choice of the specific joints treatments (hip, knee, etc.) to deliver to OA affected patients, especially in an early stage of the pathology or for sports related trauma.

## Data Availability

The raw data supporting the conclusions of this article will be made available by the authors, without undue reservation.
