# Supplementary material for: Biomechanical and biological features of hyaluronic acid in combination with chondroitin and platelet rich plasma for regenerative medicine applications
Source: Front Bioeng Biotechnol. 2025 Oct 7;13:1607469. doi: 10.3389/fbioe.2025.1607469 (PMC12537726; doi:10.3389/fbioe.2025.1607469)
Supplement: Supplementary file 1 [file DataSheet1.pdf]

Supplementary files

- **Figure S.1**

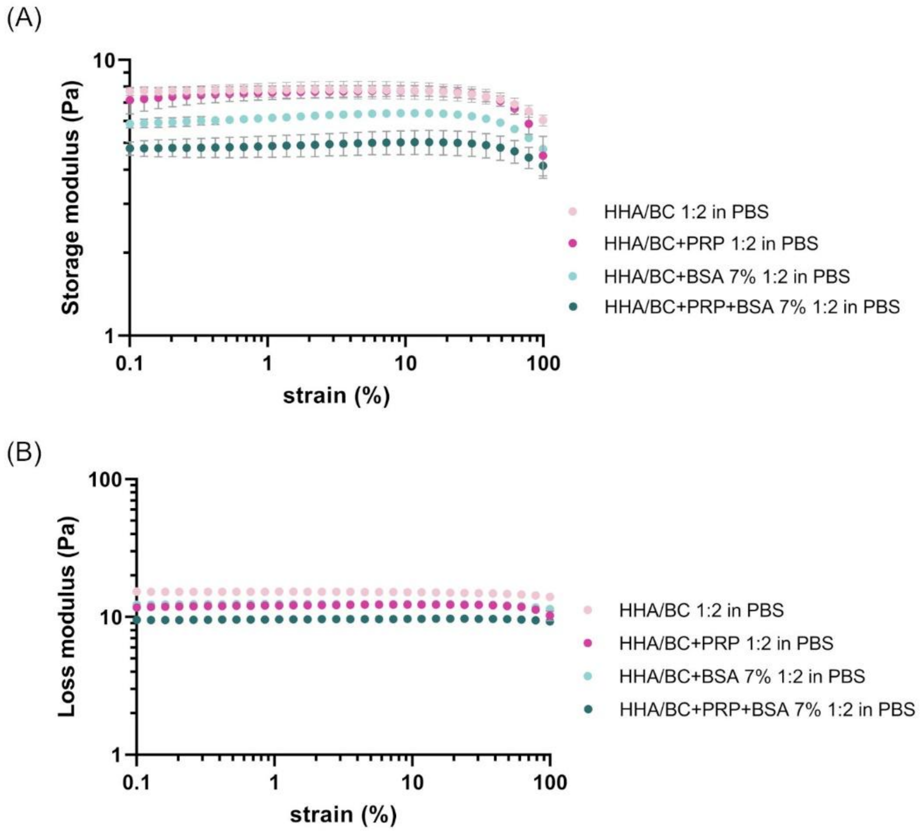

Figure S.1: Dynamic moduli as a function of deformation from 0.1 to 100% at 1.59 Hz and 37 °C: (**A**) Storage modulus G'; (**B**) loss modulus G''.
